# Visualization of large influenza virus sequence datasets using adaptively aggregated trees with sampling-based subscale representation

**DOI:** 10.1186/1471-2105-9-237

**Published:** 2008-05-16

**Authors:** Leonid Zaslavsky, Yiming Bao, Tatiana A Tatusova

**Affiliations:** 1 National Center for Biotechnology Information, National Library of Medicine, National Institutes of Health, Bethesda, MD 20894, USA

## Abstract

**Background:**

With the amount of influenza genome sequence data growing rapidly, researchers need machine assistance in selecting datasets and exploring the data. Enhanced visualization tools are required to represent results of the exploratory analysis on the web in an easy-to-comprehend form and to facilitate convenient information retrieval.

**Results:**

We developed an approach to visualize large phylogenetic trees in an aggregated form with a special representation of subscale details. The initial aggregated tree representation is built with a level of resolution automatically selected to fit into the available screen space, with terminal groups selected based on sequence similarity. The default aggregated representation can be refined by users interactively.

Structure and data variability within terminal groups are displayed using small trees that have the same vertical size as the text annotation of the group. These subscale representations are calculated using systematic sampling from the corresponding terminal group. The aggregated tree containing terminal groups can be annotated using aggregation of structured metadata, such as seasonal distribution, geographic locations, etc.

**Availability:**

The algorithms are implemented in JavaScript within the NCBI Influenza Virus Resource [1].

## Background

Interactive analysis of large amounts of data using web resources requires specialized visualization tools for representing the results of the analysis in an easy-to-comprehend form that allows convenient manipulation of the data. With the amount of influenza genome sequence data growing rapidly, researchers need machine assistance in selecting datasets and mining the data by looking into sequence similarity as well as metadata. The number of influenza virus sequences available in public databases is rapidly increasing due to collaborative genome sequencing efforts [2,3]. The National Center for Biotechnology Information (NCBI) has developed the Influenza Virus Resource, which provides public access to influenza sequence data and a convenient interface for constructing and viewing multiple sequence alignments and phylogenetic and clustering trees, as well as performing other data analyses [4]. The visualization approaches used in earlier releases of the NCBI Influenza Virus Resource were based on a sequence-level representation of the data. They provided a convenient interface for viewing the entire dataset using multiple sequence alignments and trees built using various algorithms. However, manipulating individual sequences was not very efficient for large datasets. Detailed schematic representations of a large dataset with a fine level of detail are very hard to comprehend. The problem with such representations is the inclusion of all information regardless of relevance [5,6]. The user needs guidance to scan through a complex set of data. It is much more convenient for a user to work with a representation that is adapted to the specific task. In the case of sequence datasets, the information could be shown not only at the level of individual sequences but also groups of sequences, depending on the task. Frequently, it is desirable to structure the dataset and provide meaningful aggregated representations with an ability to adapt the aggregation level. We have enhanced the tree representation in the Influenza Virus Resource in that direction.

Different aspects of data representation by trees have been widely discussed in literature. Several tree visualization systems have been developed to support interactive tree browsing with zooming ability ([7-10]). The issues of scalability, performance and robustness of tree visualization [11], exploration of complex trees and tree pattern matching [12], dynamic graphics and annotation of trees [13], and handling complexity through abstraction [14] have been discussed in relation to various applications. The problem of labeling a tree at low magnification has been approached in PhyloWidget [15] by setting the minimum text size and using a so-called competitive occlusion process.

Our approach to adaptive tree representation is also inspired by map visualization technologies [16]. Geographic information systems (GIS) widely used in mobile devices provide adaptively-coarsened visual representations of maps changing in real time to provide the best visualization suiting a specific task. They fulfill their task by serving necessary information, with knowledge being represented in an easy-to-comprehend form and the amount of information is limited in a way that a human (driver) can process it and make a reasonable decision in real time.

## Results

The approach presented in this paper allows the display of a large tree in an aggregated form with special representation of subscale details, while aggregating structured metadata consistently with tree aggregation. We presented the initial results at the ISBRA 2007 symposium [17]. This paper describes the method in more detail and discusses recent algorithm enhancements.

In our method, aggregated tree representation is calculated from the full phylogenetic tree, and terminal groups are created based on sequence similarity with the degree of aggregation determined by the amount of available screen space. Structure and data variability within terminal groups are displayed using a special *subscale representation *by a small tree that has the same vertical size as the textual annotation of the group and that is constructed using systematic sampling from the terminal group. The terminal groups are annotated using aggregation of structured metadata, such as seasonal distribution. This representation can be refined interactively. Datasets represented by trees can also be searched using both structured and unstructured metadata, including sequence names. The search results are shown as individual sequences, when resolved, or otherwise, as number of sequences in named groups satisfying the search criteria. An improved algorithm utilizes systematic sampling from the terminal group for building a subscale representation: a set of well-scattered leaves is identified, and the corresponding subtree is extracted from the full tree. This allows a more accurate and unbiased subscale-resolution representation of terminal groups than the technique we presented at ISBRA 2007 [17].

**Figure 1:**
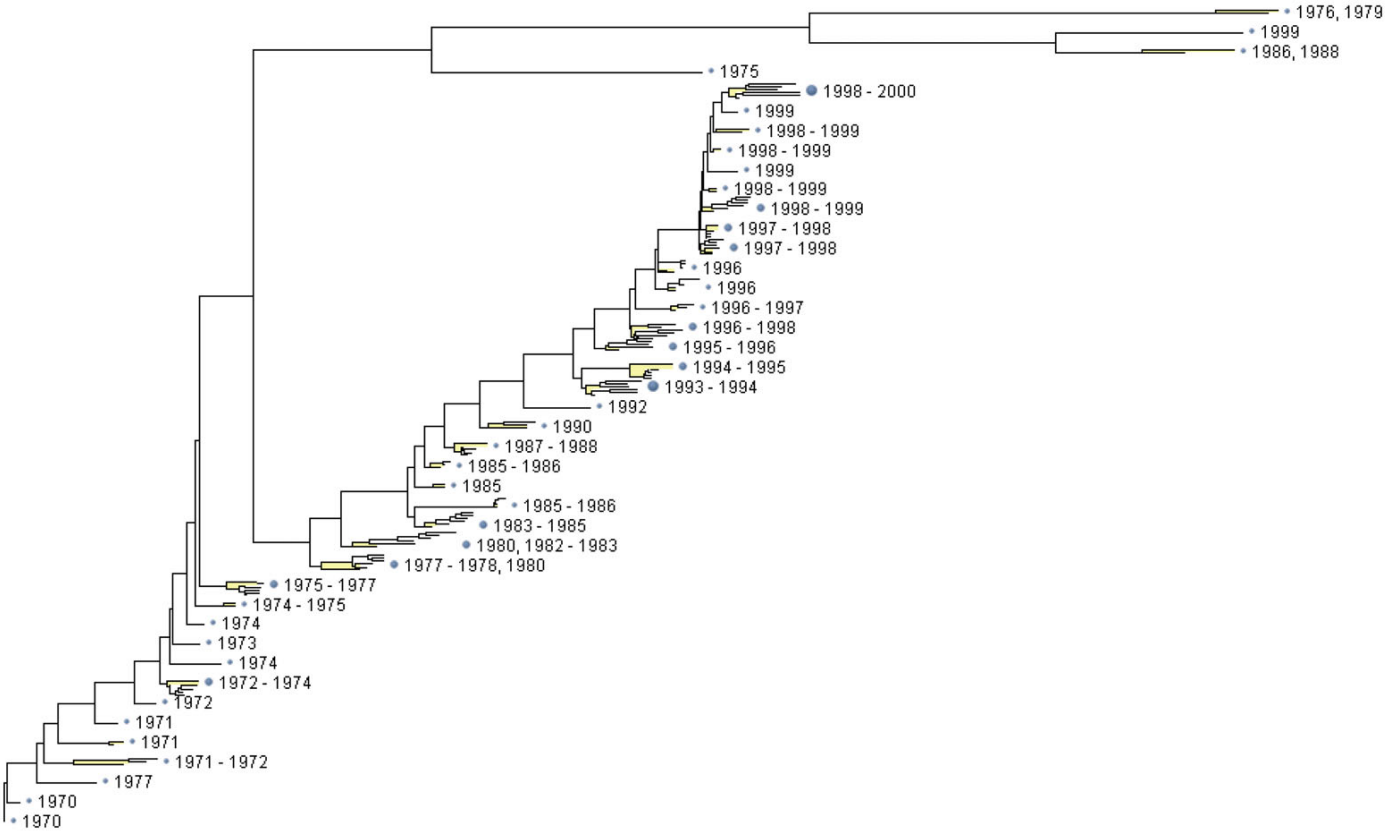
**Aggregated tree built for a dataset containing 375 HA protein coding sequences for Influenza A H3N2 viruses extracted from human hosts during a 30-year period 1970–2000.** The full tree has been built using F84 distance and the neighbor-joining method.

**Figure 2:**
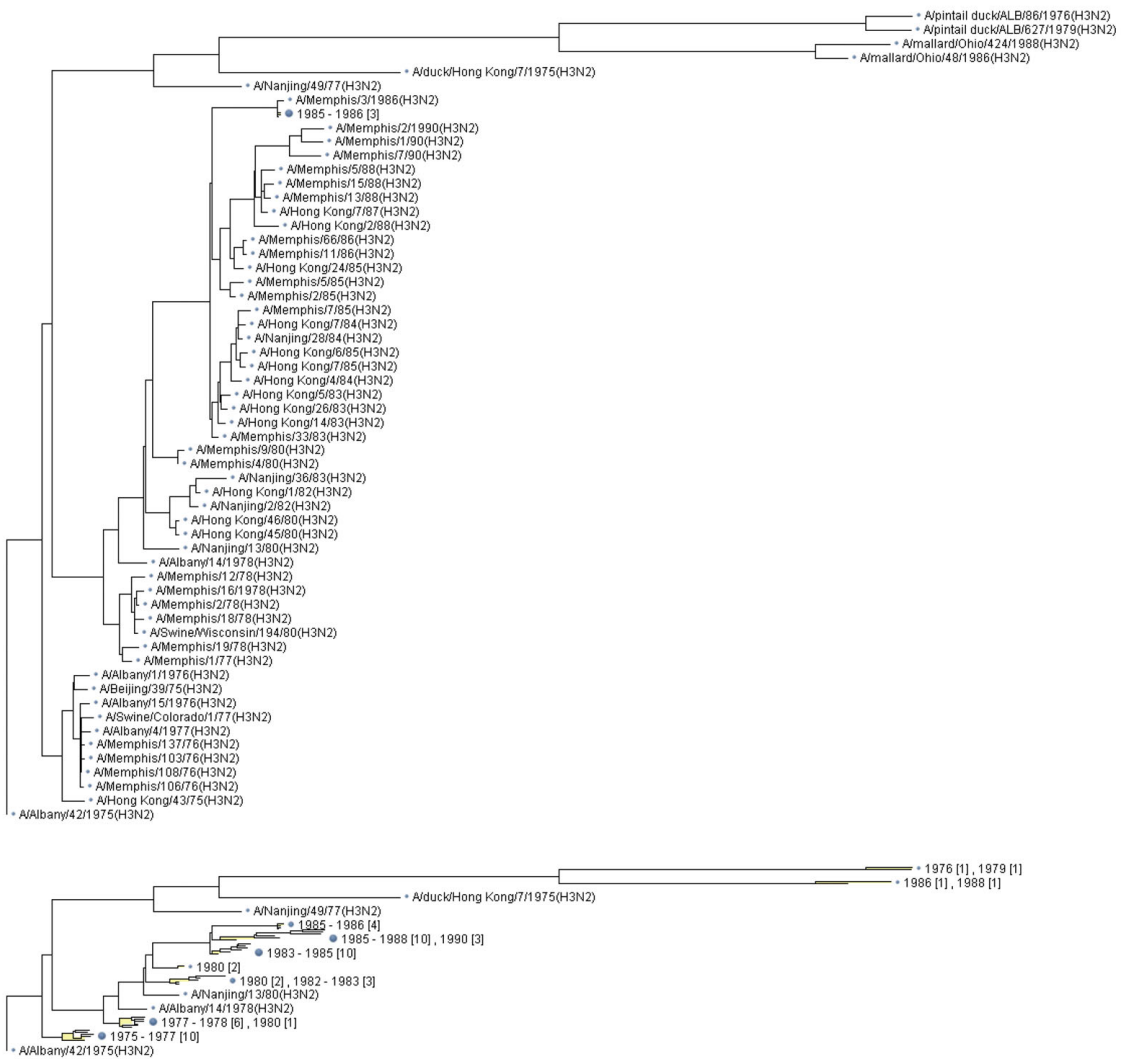
A full-resolution tree (top) and aggregated tree (bottom) built for 60 HA protein coding sequences for Influenza A H3N2 viruses extracted from human hosts during a 15-year period 1970–1985 (we used F84 distance and the neighbor-joining method).

Figures 1 and 2 illustrate usage of the method for displaying influenza virus sequence datasets. Figure 1 shows an aggregated tree with subscale representation of terminal groups for a hemagglutinin dataset containing 375 sequences from 1970–2000. Obviously, this dataset could not be displayed with full resolution on the page and on the screen. One can compare a full resolution tree and an aggregated tree shown in Figure 2 for a hemagglutinin dataset from 1970–1985.

### Aggregated tree representation

We propose a new algorithm for constructing an aggregated tree representation for a given phylogenetic tree. To build an aggregation representation, terminal subtrees that would be represented in less detail are selected. To identify terminal subtrees and control visual representation, we assign *status *values to tree nodes of the full tree. Status values provide guidance for tree visualization. The status value *s*(*i*) is assigned to each node *i *of the full tree: setting *s*(*i*) = 1 if node *i *is the root of the terminal subtree, or *s*(*i*) = 0 otherwise.

The algorithm works as follows. We start with all leaves assigned to one group, e.g., tree fully collapsed, and perform disaggregation up to available screen space. Technically, disaggregating node *i *results in setting the status value of the node to 0 and the status values of its children to 1. The nodes are disaggregated starting from the root of the tree *r*. At each step, a node with the largest diameter^1 ^of the corresponding subtree is chosen for disaggregation among available candidates. To control the order of node disaggregation, we use a *max*-priority queue Θ. It contains records *R*_*k *_= (*i*_*k*_, $d_{i_{k}}$), where *i*_*k *_is the index of the tree node, $d_{i_{k}}$ is the diameter of the corresponding subtree, and *k *= 0, 1, 2,... . The front element of the priority queue *R*_0 _= (*i*_0_, $d_{i_{0}}$) has the maximal diameter of the subtree. Our current implementation of the priority queue Θ utilizes a JavaScript Array object, where records are kept sorted by non-increasing subtree diameters: $d_{i_{k}}$ ≥ $d_{i_{m}}$ for any *k *<*m*, 0 ≤ *k*, *m *< |Θ|. A binary search is performed to find the insertion position for each new record, while the JavaScript method Array::splice is used for inserting a record in the array.

Denote the maximal number of terminal groups allowed as *N*_*max*_, and the current number of groups in the aggregated tree as *N*. Let Λ_*i *_be the set of children of node *i*. The disaggregation algorithm can be formally described as follows.

**ALGORITHM 1**. Building an aggregated tree

**Set root status ***s*(*r*) = 1;

**Set*** N ***to** 1.

**Include root ***r ***in the ***max*-**priority queue **Θ;

**While **(|Θ| > 0 **and ***N *+ max(|$\Lambda_{i_{0}}$| - 1, 0) ≤ *N*_*max *_**) {**

   **Set ***s*(*i*_0_) = 0, **where ***i*_0 _**is the node index of the front element of **Θ;

   **If ($\Lambda_{i_{0}}$ ≠ ∅ ){**

      **For (all ***k *∈ $\Lambda_{i_{0}}$**){**

         **Include ***k ***in **Θ;

         **Set ***s*(*k*) = 1;

      **}**

      **Set ***N *← *N *+ |$\Lambda_{i_{0}}$| - 1.

   **}**

   **Remove the front record from the ***max***-priority queue **Θ;

}

^1^Diameter of the subtree is defined as the maximum of tree distances between subtree leaves. In turn, distance between two tree nodes is defined as the length of the shortest path between them.

### Building subscale representations for terminal groups

Each terminal group is shown using a single-line text annotation and a small tree occupying the same vertical size as the text annotation, which we call *subscale representation*. While many details of the subtree structure for the terminal group are abandoned, branch length variation within the group and overall structure of the group are displayed (see Figures 1 and 2).

In this section we present a new algorithm for building subscale representations. An earlier algorithm that we presented at ISBRA 2007 [17] was similar to Algorithm 1: it started with a single group, and several cycles of disaggregation were performed until all available vertical space was utilized. Unresolved groups of nodes were shown by their shortest and longest branches. This earlier algorithm allowed visualization of the branch length variability in terminal groups and group structure. However, it did not always represent the structures of large terminal groups accurately. Particularly, we observed good representation of balanced terminal subtrees, while poor representation of unbalanced terminal trees was due to biased sampling. The new algorithm described below utilizes systematic sampling from the terminal group. First, a set of well-scattered leaves is found among the elements of the terminal group, then a tree with leaves consisting of the elements of the selected set is extracted from the full subtree corresponding to the terminal group. Because of the explicit sampling, the new algorithm avoids the bias problem and allows a much more accurate and meaningful subscale representation. We propose selecting a representative set of leaves from the terminal group by systematic sampling [18]. When a set of well-scattered leaves is found in the terminal subtree, we select a tree spanned by them and the subtree root.

If the tree is binary, and the vertical size of the subscale subtree is approximately *N*_*vrt *_pixels, then the maximal number of leaves *N*_*l *_in the subscale tree is *N*_*l *_= [(*N*_*vrt *_+ 1)/2]. Let *d*^*T *^(*x*, *y*) be the length of the path between nodes *x *and *y *(also known as tree distance between the nodes), and *d*^*T*^(*x*, *S*) be a tree distance between node *x *and set of nodes *S *defined as

*d*^*T*^(*x*, *S*) = *min*_*ν*∈*S*_*d*^*T*^(*x*, *ν*).

Let *F *be a set of leaves of the subtree. The algorithm computes a set of well-distributed nodes *M *⊆ *F *of size *N*_*l*_. Without loss of generality we can assume that |*F*| ≥ 2 and *N*_*l *_≥ 2.

**ALGORITHM 2**. Systematic sampling leaves of the subtree

**Find ***x*_- _∈ *F ***closest to the root of the subtree;**

**Find ***x*_+ _∈ *F ***furthest from the root of the subtree;**

**Set ***M *= {*x*_-_, *x*_+_} **and **Λ = *F *\ {*x*_-_, *x*_+_}.

**While ( **Λ ≠ ∅ **) {**

   **Select an ***ζ *= arg max_*ν*∈Λ _*d*^*T*^(*ν*, *M*);

   **Move ***ζ ***from **Λ **to*** M*.

}

Each time a new element *η *is included in set *M*, the value of the distance from each remaining element *x *to set *M *is updated as follows:

*d*^*T*^(*x, M*) = min(*d*^*T*^(*x*, *η*), *d*^*T*^(*x*, *M*_0_)),

where *x *∈ *F*, *M*_0 _⊆ *F η *∈ *F *and *M *= *M*_0 _∪ {*η*}.

**Figure 3:**
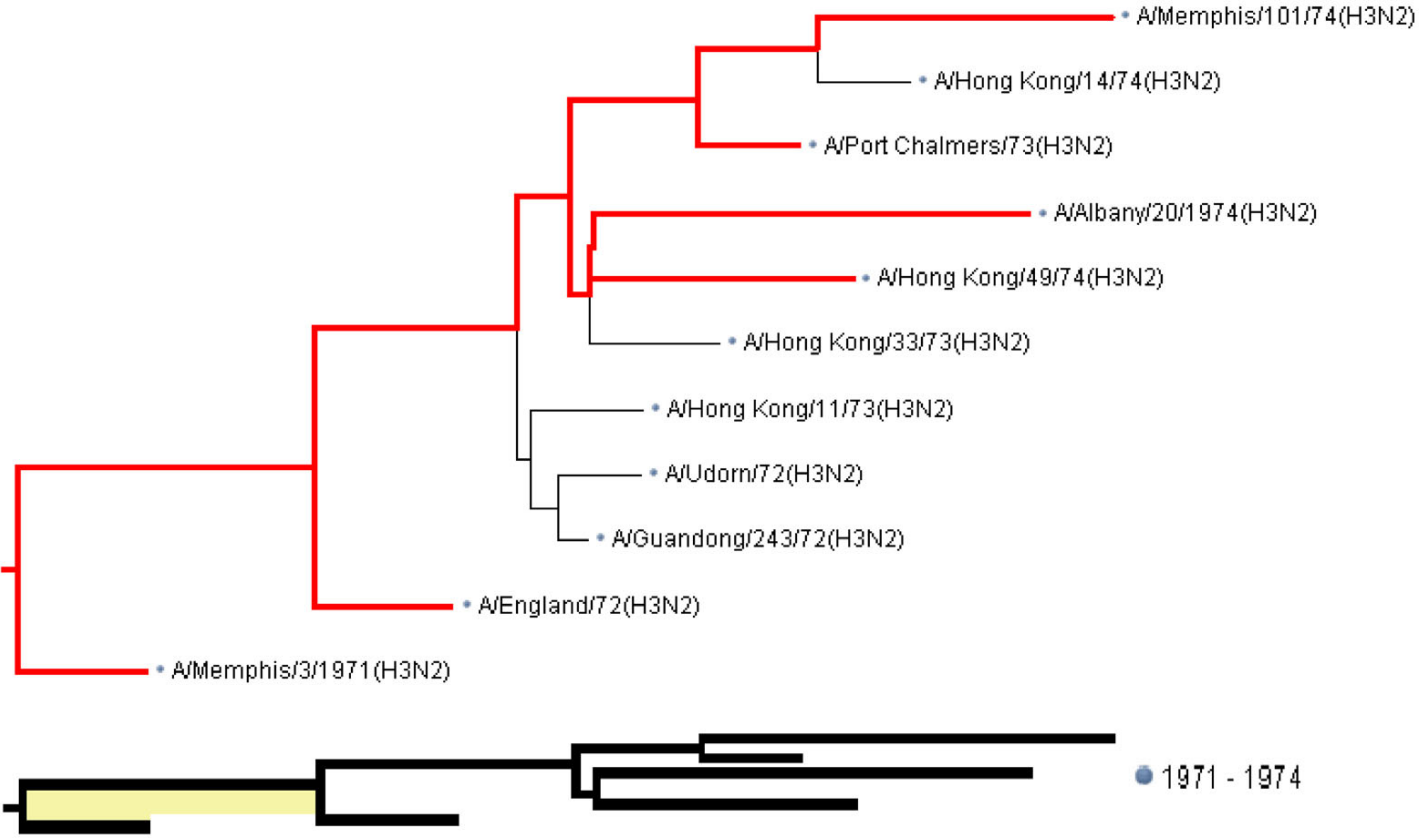
**A subtree of the phylogenetic tree in Figure 1 shown in full resolution (top) and its subscale representation (bottom).** The tree spanned by sampled leaves and the root is shown in red color.

Figure 3 shows a subtree of a phylogenetic tree and a subscale representation of that subtree built using a systematic sample of its leaves.

### Terminal group annotation using aggregated structural metadata

Aggregated groups of sequences need abstracted descriptions for tree annotation. It is possible to summarize the group using descriptive characteristics: virus type, subtype, year of extraction, season of extraction, geographical location (country, continent). Aggregation by year is shown in Figures 1 and 2). However, abstracting or summarizing less formal descriptions, such as strain name, is more challenging [19].

### Implementation and availability

The algorithms described in this paper are implemented using JavaScript and work on the client site. They are part of the new AJAX-based implementation of the NCBI Influenza Virus Resource [1]. Information about service availability and access to the code at the NCBI is provided separately [see Additional file 1].

## Discussion

Influenza A viruses are known to exhibit primarily directed evolution, with small lineages branching out and dying, and new major lineages rarely appearing. An attentive observer will find even smaller lineages branching out from minor branches and "dying" faster (in mathematical terms, a typical influenza dataset has a very low estimated value of Kolmogorov dimension, also known as box-counting dimension [20]). The seemingly linear character of influenza evolution inspired scientists and practitioners to look for methodologies to predict a major influenza strain using past data (see [21] and references therein). However, long intervals of slow linear change are interrupted by short intervals of rapid change [22]. The low dimensionality of structure of typical influenza datasets and their multiscale properties allow the use of the aggregated representation described above. Our aggregated representation design makes it possible to focus on important properties of the dataset, and in particular, adapt to the speed of change. This is due to the choice of criteria to prioritize node disaggregation and the choice of sampling method for unresolved terminal groups. At both stages, the decision is based on "importance" as measured by sequence diversity (maximal diameter of the group in disaggregation; well-distributed set in sampling, with distribution measured by distance). Our importance-based approach allows automatic adaptation to the speed of change: time intervals with rapid change are resolved in greater detail than the ones with slow change. Further refinements can be conveniently performed by the user.

Note that the importance-based systematic sampling technique for constructing subscale-resolution representations of the terminal groups that is used in this approach could also be used for reducing the dataset in multiple sequence alignments and in bootstrap analysis. From a computational point of view, it may be feasible to perform sampling using approximate information provided for the dataset (say, by BLAST), and perform more costly multiple sequence alignments only for a sample. It can also be used to reduce the dataset in a bootstrap analysis used for building a consensus tree for the dataset, since bootstraping requires computing multiple trees from randomized distance matrices [23-25]. The user can use a computer-generated systematic sample directly or correct it manually.

## Conclusion

Adaptive aggregated trees provide a convenient way of representing the results of a preliminary analysis of large sequence datasets and enables the user to manipulate the data hierarchically, performing each operation at the appropriate resolution level. Subscale representation allows the display of an overall structure and branch length variability within terminal group. A new algorithm for building subscale representations based on the systematic sampling from the terminal groups allows an unbiased subscale resolution representation of the group.

## Authors' contributions

LZ proposed, designed, implemented, tested and evaluated the method, and wrote the manuscript. YB and TAT participated in the design and evaluation of the method, and contributed to the paper. TAT is the technical lead for the NCBI Influenza Virus Resource project. All authors read, made corrections and approved the final manuscript.

## Supplementary Material

Additional file 1 Service availability and locations of source files. Information about service availability and access to the code at the NCBI. Click here for file
