# Supplementary material for: Visualization of large influenza virus sequence datasets using adaptively aggregated trees with sampling-based subscale representation
Source: BMC Bioinformatics. 2008 May 16;9:237. doi: 10.1186/1471-2105-9-237 (PMC2416652; doi:10.1186/1471-2105-9-237)
Supplement: Additional file 1 — Service availability and locations of source files. Information about service availability and access to the code at the NCBI. [file 1471-2105-9-237-S1.html]

   Service availability and locations of source files    

|  |
| --- |
|  |
| **Service availability and locations of source files** |
| The algorithms described in this paper have been implemented in JavaScript within The NCBI Influenza Virus Resource, a free web service provided by the National Center for Biotechnology Information (NCBI). |
| The current implementation can be seen in the files treeStructureLib.js and treeGraphicsLib.js that can be accessed at the NCBI web site under the terms of The NCBI Copyright Statement, Disclaimers and Conditions of Use. This software is a "United States Government Work" under the terms of the United States Copyright Act. It was written as part of the official duties of authors as United States Government employees. |
| The access to the source code is granted solely for the purpose of demonstrating our implementation of the methodology described in the paper. The code has been designed to serve within the NCBI environment as a part of the NCBI Influenza Virus Resource and has not been tested in any other environment or for any other purpose. |
